# Exploring the influence of residential courtyard space landscape elements on people's emotional health in an immersive virtual environment

**DOI:** 10.3389/fpubh.2022.1017993

**Published:** 2022-10-28

**Authors:** Erkang Fu, Jiawen Zhou, Yuxin Ren, Xiaoyu Deng, Lin Li, Xinyun Li, Xi Li

**Affiliations:** College of Landscape Architecture, Sichuan Agricultural University, Chengdu, China

**Keywords:** virtual reality technology, residential courtyard space, environmental elements, emotional health, environmental health

## Abstract

A good residential courtyard space not only brings people a psychological feeling of emotional pleasure but also attracts people to actively engage in more physical activities, which is of great significance to improving people's physical and mental health. Green vegetation and fitness facilities as the most preferred landscape elements in residential courtyard spaces and their effects on people's emotional health have not been further investigated. In this research, a virtual experiment was constructed in a residential courtyard space in Chengdu City, China, and electrodermal sensors were used to measure the real-time emotional changes of people in different virtual scenarios to analyze the effects of two landscape elements, green vegetation and fitness facilities, on people's emotional health, and the differences of such effects among different genders and ages. The results confirm that the combination of virtual reality technology and wearable physiological sensor measurement technology could effectively identify health-promoting landscape elements in urban environments; In residential courtyard spaces, green vegetation is more emotionally healthy than fitness facilities, and fitness facilities have better emotional health benefits for men and people over 30 years of age. The results of the study provide a quantitative basis for the healthier design and transformation of residential courtyard spaces for both green vegetation and fitness facilities.

## Introduction

Rapid urbanization has given rise to a range of human health problems, such as obesity, stress overload, depression, cardiovascular disease, burnout syndrome and neurological, and immunological disorders (1). In 1983, Stephen Kaplan and Talbot, professors of psychology at the University of Michigan, introduced the concept of “restorative environments,” which is defined as “environments that enable people to better recover from mental fatigue and the negative emotions associated with stress” (2).

In recent years, more empirical studies have been conducted in academia on factors in urban public spaces that directly or indirectly affect the emotional health of populations. Kisteman et al. found that public spaces with waterfront environments were effective for human health and stress reduction (3), and Deli et al. compared the restorative benefits of water spaces, wooded spaces, and lawn spaces in urban parks by collecting different physiological indicators (4). Karin found a relationship between the potential health uses of pocket parks and spatial elements such as park shape and size (5). Dong and Pan pointed out that the comfort of physical conditions such as light and site leveling is the primary condition for the community public space that affects the choice of healthy activities for the elderly (6), Tan et al. have identified urban transport, recreation, built and green environments based on active health interventions for walking behavior (7). Meanwhile, previous studies point out that environmental elements that promote people's emotional health include naturalness, perception, rest, service, safety, and activity. Among the natural elements, green vegetation is considered to be the most important landscape element (8–11), and the green view rate is considered to be the best indicator of environmental effects on stress reduction and emotional recovery (20–40% decompression effect is the best) (12). Xu et al. demonstrated through VR experiments that the green view rate and openness of street interface have significant effects on street healing (13), and Fu Erkang et al. also found that a high green view rate and low openness could effectively promote the perception of healing in street space in a study of audio-visual interactive street healing environment (14). In addition, positively valued man-made features play a key role in the visual quality of the environment and are an important landscape element (15). Heinen et al. found that an abundance of street furniture settings was more likely to create safe walking and cycling environments, thereby increasing the number of people engaged in daily physical activity, while Liao and Wang study further illustrates that good outdoor fitness facilities can also stimulate people to engage in The study by Liao and Wang further suggests that good outdoor fitness facilities can also stimulate physical activity and thus affect emotional well-being (16, 17).

As the outdoor living room of people's daily life, the environment of Residential Courtyard Space could have a direct impact on the physical and mental health of residents (18). Hartig T et al. used an exercise feasibility study to verify the effects of urban residential environments and community park environments on the emotional health of different occupational groups (19); Kuo et al. compared the effects of different types of outdoor natural environments in residential areas on people's physical and mental health and found that environments with distant trees improved cognitive performance more than close grassy areas (20); Tennessen et al. found that young people who could see nature through their dormitory windows performed better on tasks that required concentration (21); Dong and Pan et al. found that the comfort of physical conditions, such as light and site level, was the primary condition that influenced older people's choice of community public spaces for healthy activities (6). These studies have led to the finding that, on the one hand, the greenery in Residential Courtyard Space, as the most accessible natural environment for the residents in their daily lives, has a positive effect in alleviating the sense of stress and fatigue and promoting the sense of vitality of the residents, thus promoting their emotional well-being (22); on the other hand, the appropriate sports facilities in residential courtyard space can attract the residents to engage in more physical activities, which in turn has a certain mood-boosting effect (23). However, while there is much evidence that planning and design interventions to improve the visualization of nature and environmental amenities in residential courtyard spaces can be effective in alleviating stress responses and enhancing cognitive abilities, thereby improving physical and mental health, it is a challenge to quickly identify the landscape elements that affect emotional well-being and their differential impact on different populations in practical planning and design (12, 24).

Virtual Reality (VR) is a computer technology as the core, generate and a certain range of real environment in the visual, auditory, tactile, and other aspects of similar digital environment (25), because of a good sense of immersion, interaction, and creation and become a new way to experience the urban environment (26), has been in the architecture and interior of the auxiliary design (27, 28), the virtual design of cultural heritage (29), the virtual urban environment display (30, 31), spatial cognition research (32, 33), etc. As a new interactive medium, VR technology constructs urban spaces with different environmental characteristics that could help urban designers effectively identify those landscape elements that may have emotional health-promoting effects in order to achieve health goals when designing and renovating, and updating spaces. The Centre for Emotion Research of the US Department of Mental Health has found through extensive experiments with pictorial emotional stimuli that subtle physiological responses to human emotions can be measured more accurately and quickly by biosensors (34). Chen et al. from Tongji University in China relied on experimental methods to record human emotional experiences during real-time environmental walking through skin conductance sensors, etc. The emotional results collected by the sensors matched well with the results of environmental experiences expressed by the subjects in post-test interviews in the field, confirming that physiological, emotional data from spatio-temporal trajectories in urban environments can better reflect the subjects' spatially influenced emotional responses (35). Xu et al. simulated two different urban environments, roads, and green spaces, in a VR system, and the collected electrodermal data indicated that green spaces had better healing properties than road landscapes. Therefore, the simultaneous collection of real-time emotional data from experiencers in experimental scenarios constructed by VR technology is a faster and more accurate way to reflect people's subjective feelings toward the environment and to effectively control the variables.

This paper designed a VR control experiment based on the residential compound space of No. 12, Dongli Community, Chengdu City, and conducted a quantitative study using wearable electrodermal sensors to try to analyze the effects of residential compound space landscape elements on the emotional health of the population. Specifically, there are three research questions: (1) Are there any differences in the effects of two landscape elements, green vegetation and fitness facilities, on the emotional health of the population in the residential compound space? (2) Are there gender differences in the emotional health benefits of the two landscape elements of green vegetation and construction facilities? (3) Are there any age differences in the emotional health benefits of the two landscape elements of green vegetation and construction facilities?

## Methods

### Virtual experiment scenario construction

**Figure 1:**
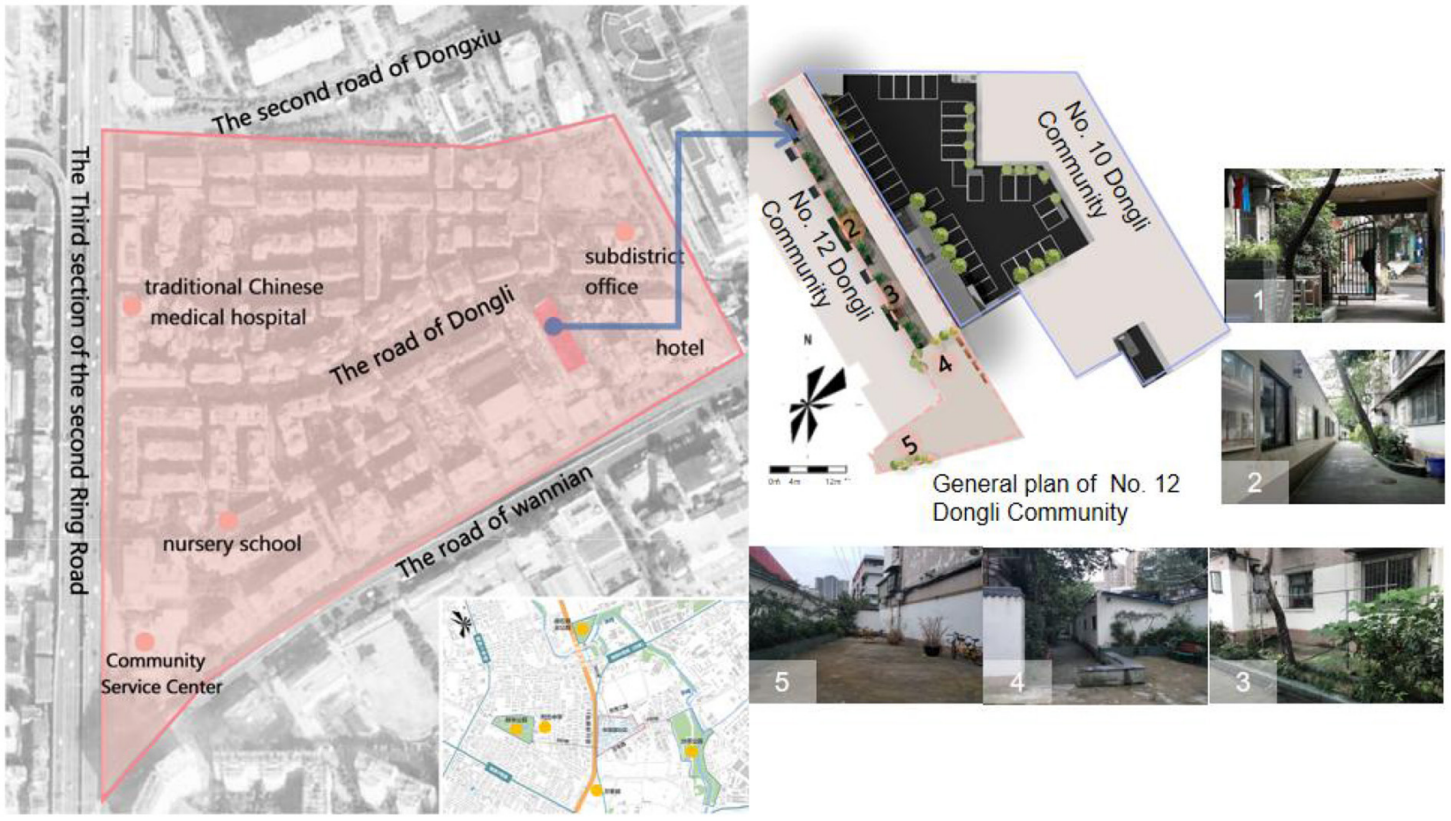
Overview of the current situation at No. 12 Dongli Community.

In this study, a sample space was selected for the construction of a virtual experimental scenario in No. 12 Dongli Community. The site is located in the centre of Chengdu, a crucial central city in West China chosen by the State Council (36). The Dongli community is typical of old urban settlements, with the main road running east-west through 21 interlocking residential courtyards, and complete facilities such as hospitals, kindergartens and street green areas, which can basically meet the daily needs of residents. Among them, No. 12 Dongli is a traditional courtyard of mainly indigenous residents, with the elderly and children as the main population, its narrow and long spatial pattern, single environmental elements, lack of green vegetation and sports and fitness facilities, and ineffective use of existing space are typical of a living environment lacking in healthy design (Figure 1).

**Figure 2:**
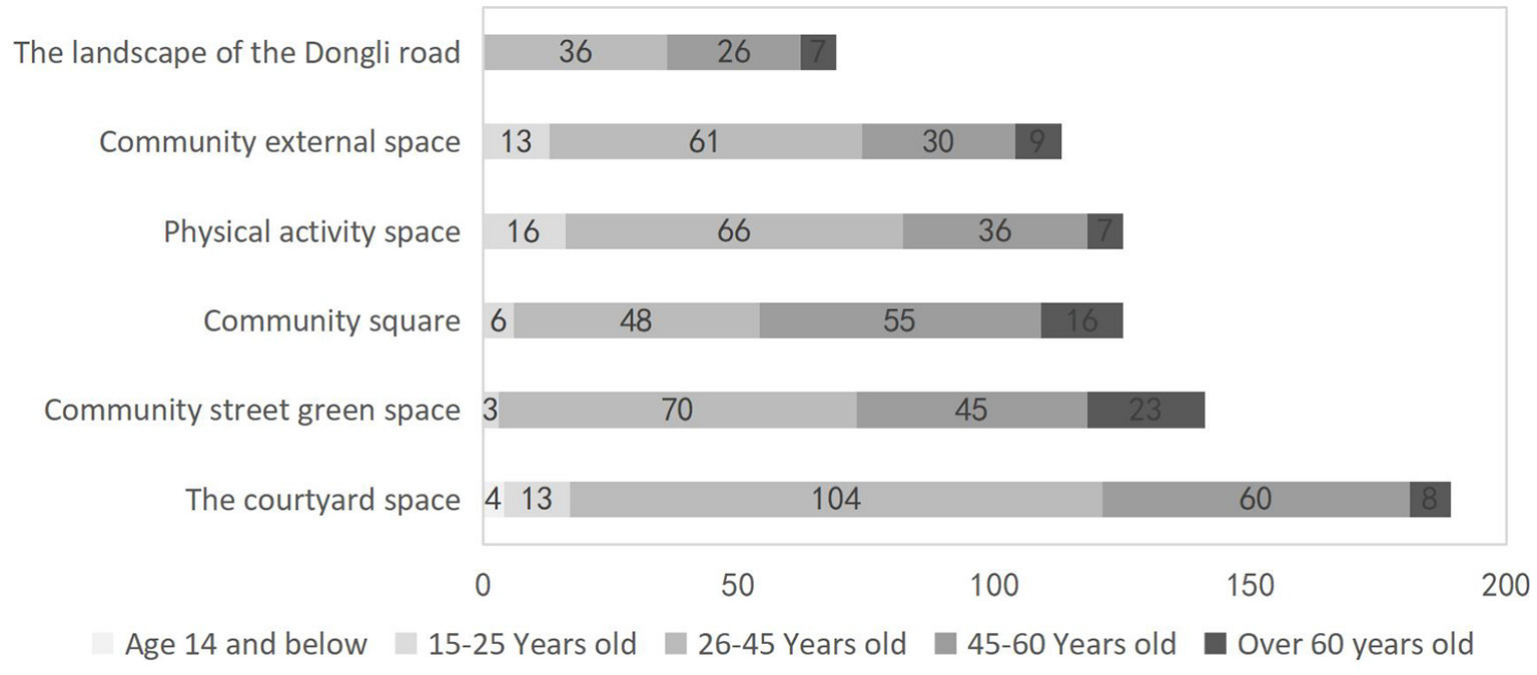
Survey on outdoor space preference of residents in Dongli community.

**Figure 3:**
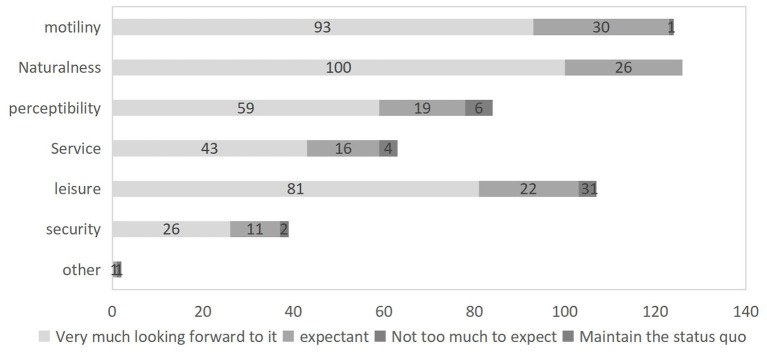
Survey on the willingness of residents of Dongli community to transform courtyard space.

Research in the field of environmental psychology has shown that a reasonable configuration of landscape elements such as nature, activity, rest and service could alleviate the psychological stress and bad mood of people, promote more physical activity and thus influence their psychological and physical health (37). Research in the field of environmental psychology has shown that a reasonable configuration of landscape elements such as nature, activity, rest and service can alleviate the psychological stress and bad mood of people, promote more physical activity and thus influence their psychological and physical health. In order to investigate more precisely the landscape elements of residential courtyard space that affect people's emotional health, this study conducted a survey on the willingness of 451 indigenous residents of different ages in the Dongli community to transform their courtyard space, and the results showed that “courtyard space” was the second most preferred outdoor space after “street green space” (Figure 2). “Abundant vegetation” and “provision of sports facilities” were the most popular landscape elements of courtyard space (Figure 3). Therefore, two indicators were chosen for this study, namely the green view rate, which reflects the level of greenery, and the number of sports facilities, which reflects the level of sports facility configuration. Sketchup software was used to construct the basic model of the architectural interface, fences, paving, and other environmental elements of Dongli 12, and Mars software was imported to edit the gradient of greenery and sports facilities components to construct three virtual test scenes with different environmental characteristics. As shown in Table 1, scenario A has a moderate amount of trees and irrigation with a green view rate of 40% and three sets of fitness facilities; scenario B has a moderate amount of trees and irrigation with the same green view rate of 40%, but no fitness facilities; scenario C has no trees and irrigation, a small amount of lawn with a green view rate of <10%, and the same as scenario A, with three sets of fitness facilities.

**Table 1:** Three virtual experiment scenarios.

**VR scenes**		**A**	**B**	**C**
Spatial feature	Green vegetation	Planted with a moderate amount of trees and shrubs 40% Green Vision	Planted with a moderate amount of trees and shrubs 40% Green Vision	No trees and shrubs, little lawn Green vision rate <10%
	Fitness facility	Layout of 3 sets of fitness facilities	No sports and fitness facilities	Layout of 3 sets of fitness facilities
The scenario mode		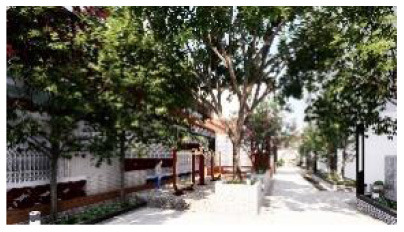	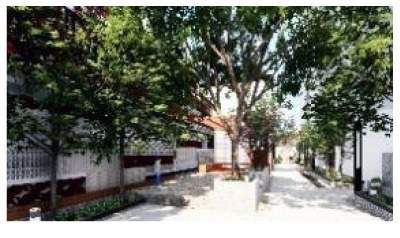	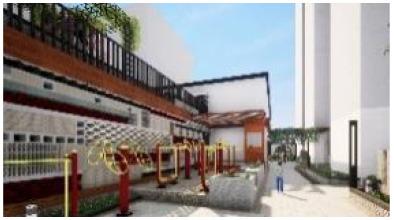

### Participants

With the help of the Dongli community committee, we recruited a total of 48 residents of the Jumping Stomp River community as subjects to participate in the experiment, including 23 males and 25 females with an average age of 47 years (Table 2). We recruited subjects who had lived in the Dongli community for more than 2 years and were familiar enough with the environmental conditions of the community. All subjects met the following conditions: (1) physically healthy, no physical or mental illness; (2) No myopia or wear contact lenses for the easier wearing of VR devices; (3) able to wear VR devices continuously for short periods of time without adverse reactions such as dizziness; (4) no undesirable hobbies (such as smoking, drug abuse); (5) no recent major changes resulting in drastic emotional changes. Prior to the start of the experiment, a gathering was organized to introduce the subjects to the procedures, voluntary nature, and confidentiality of the study and to confirm that they would have sufficient time to complete the entire experiment, asking them to avoid smoking, alcohol and strenuous physical activity throughout the study period. The study was reviewed by the Experimental Animal Ethics and Welfare Committee of Sichuan Agricultural University, China, and complied with animal protection, animal welfare and principles, and the relevant national regulations on animal welfare ethics.

**Table 2:** Information of participants.

**Gender**
Men	47.9%
Women	52.1%
**Age**
<14	18.8%
15–30	27.1%
30–55	35.3%
>55	18.8%

### Virtual reality experimentation platform

**Figure 4:**
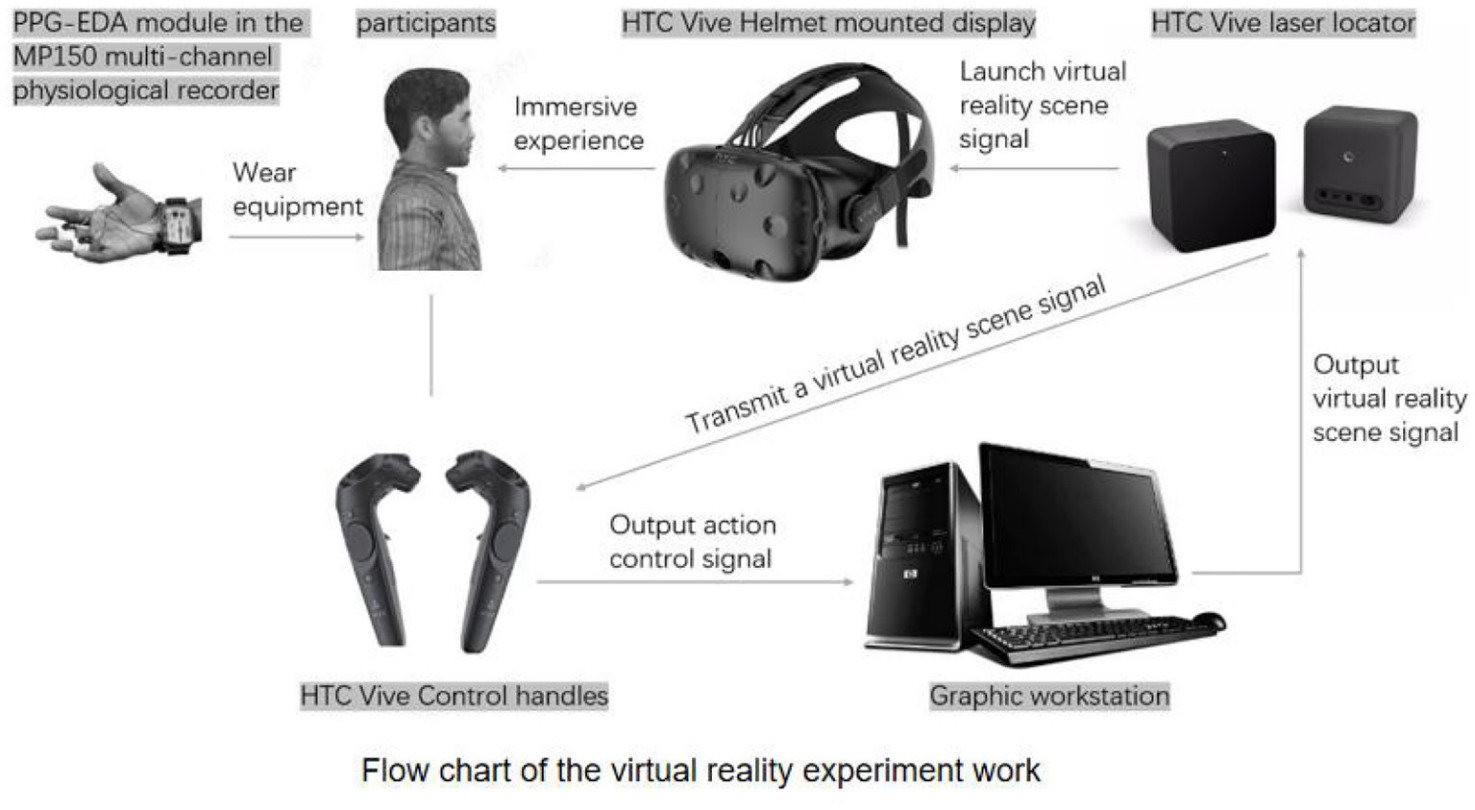
Virtual reality experiment platform workflow.

The virtual reality experiment platform of this study consists of three parts: software system, hardware system, and biometric system (the platform composition is shown in Table 3, and the workflow is shown in Figure 4. The software system is used to realize virtual scene creation and environment simulation and to control the real-time interaction behavior between human and virtual environment; the hardware system is responsible for outputting scene information to the user, creating an immersive virtual environment and also providing a window for human-computer interaction control; the biometric system is responsible for real-time monitoring of human emotional state during the interaction process.

**Table 3:** Virtual reality experiment platform.

**Subsystem**	**Functional unit**	**Usage and working principle**
Software system	Sketchup	Modeling software, generate *. skp model file
	Mars	Import the flt model file and set the scene virtual environment (such as sunshine, sky, vegetation, etc.) and finally output the virtual reality file
Hardware system	Graphic workstation	Build and run VR experimental scenes (a professional graphics card with three-dimensional signal, output and synchronization functions is required)
	HTC Vive Helmet mounted display	Enter the window of the VR experiment scene, with a sensor that can be tracked by the laser locator, and present the VR scene running on the graphics workstation to the experiencers
	HTC Vive Control handle	Responsible for the interaction between the participants and the virtual reality scenes, with a sensor that can be tracked by a laser locator
	HTC Vive Haser locator	Transmit the signal to the helmet display and control handles, and install it in the diagonal position of the room
Physiological measurement system	PPG-EDA module in the MP150 multi-channel physiological recorder	Real-time measurement of the participants' skin conductance value when experiencing the VR scenes to obtain their emotional state

### Experimental procedure

**Figure 5:**
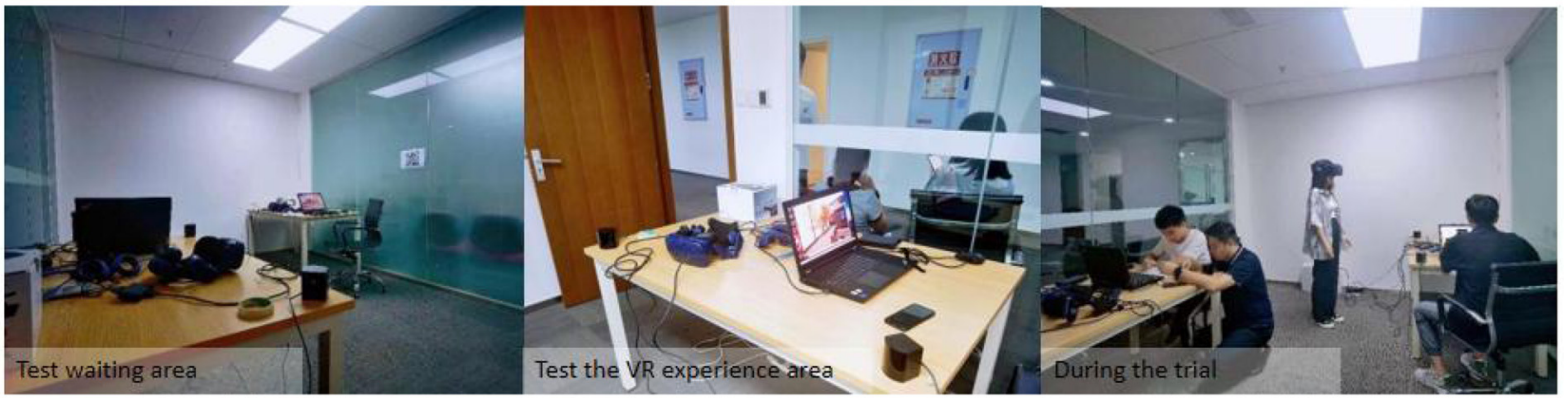
Experimental environment and process.

**Figure 6:**
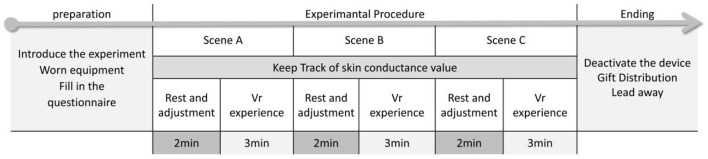
Flow chart and time distribution of single experiment.

The experiment was conducted from October 8 to October 13, 2021, with an average daily temperature of 18.7 ± 1.9°C and average daily humidity of 77.4 ± 8.6%. In order to eliminate the influence of the experiential order of the virtual experimental scenes on the subjects' emotional states, we designed the three virtual experimental scenes into six sequences of ABC, ACB, BAC, BCA, CAB, and CBA, and divided the subjects into six groups each to complete one group (i.e., one sequence) each day. The experiment was conducted in the Dongli Community Service Centre. To avoid interference from other irrelevant variables, two adjacent rooms with good ventilation were chosen to conduct the experiment, one as a waiting area and one as a VR experience area (Figure 5). Before the experiment started, the staff introduced the procedure and precautions to the subjects. Each subject followed the following procedure to conduct the experiment (Figure 6): (1) Wear the electrodermal detection equipment and sit quietly for 2 min to achieve a calm state; (2) wear the HTC VIVE virtual reality headset and handle and go to the first virtual test scene to immerse in the experience for 3 min, recording the subject's skin electrophysiological data throughout; (3) take off the headset and handle and sit still for 2 min to achieve a calm state; (4) Repeat the above two steps for the second and third VR scenes; (5) Disarm the device and guide away. The duration of the single-person sub-test was around 15 min.

### Measurement

The study used SPSS22.0 to analyse the changes in skin electrical activity before and after the VR scenes using paired *t*-tests on the acquired skin electrical data. The difference between the age and gender of the subjects in each scenario was analyzed separately using two independent samples *t*-tests.

## Result

### Effects and differences of the three virtual experimental scenarios on the emotional health of the population

**Figure 7:**
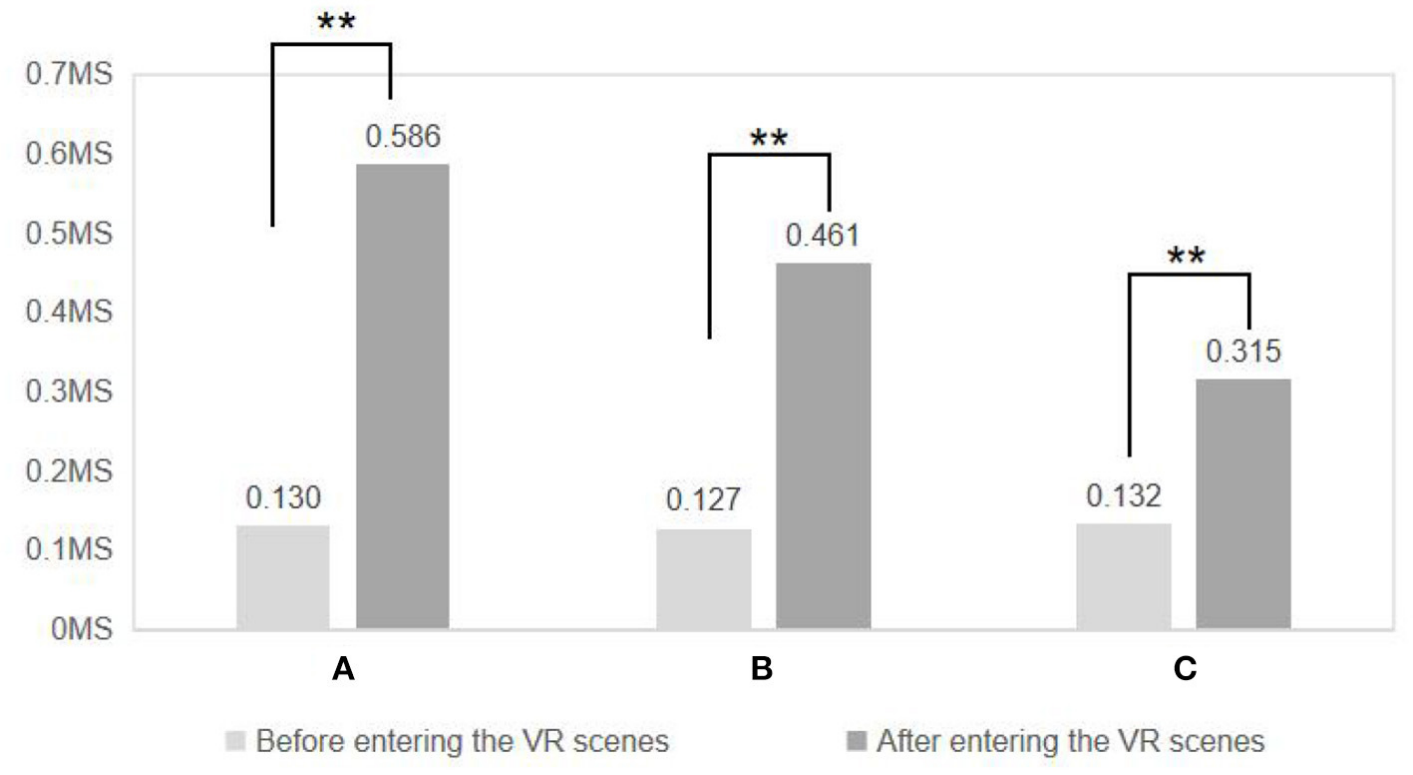
Comparison of skin electricity data after entering three VR environments. *N* = 48; mean ± SD; **P < 0.01; verified by paired *t*-test.

As shown in Figure 7, the subjects' electrical skin data changed significantly before and after the experience of the three virtual experimental scenarios (Scenario A: before 0.130 ± 0.061, after 0.586 ± 0.313, *P* < 0.01; Scenario B: before 0.127 ±0.061, after 0.461 ± 0.161, *P* < 0.01; scene C: before 0.132 ± 0.050, after 0.315 ± 0.169, *P* < 0.01). This result showed that the subjects had significantly higher skin electrical values after entering the VR scenario, confirming the equally positive effect of the spatial environment of the residential compound on the emotional health of the population in the virtual experimental scenario constructed by VR technology.

**Figure 8:**
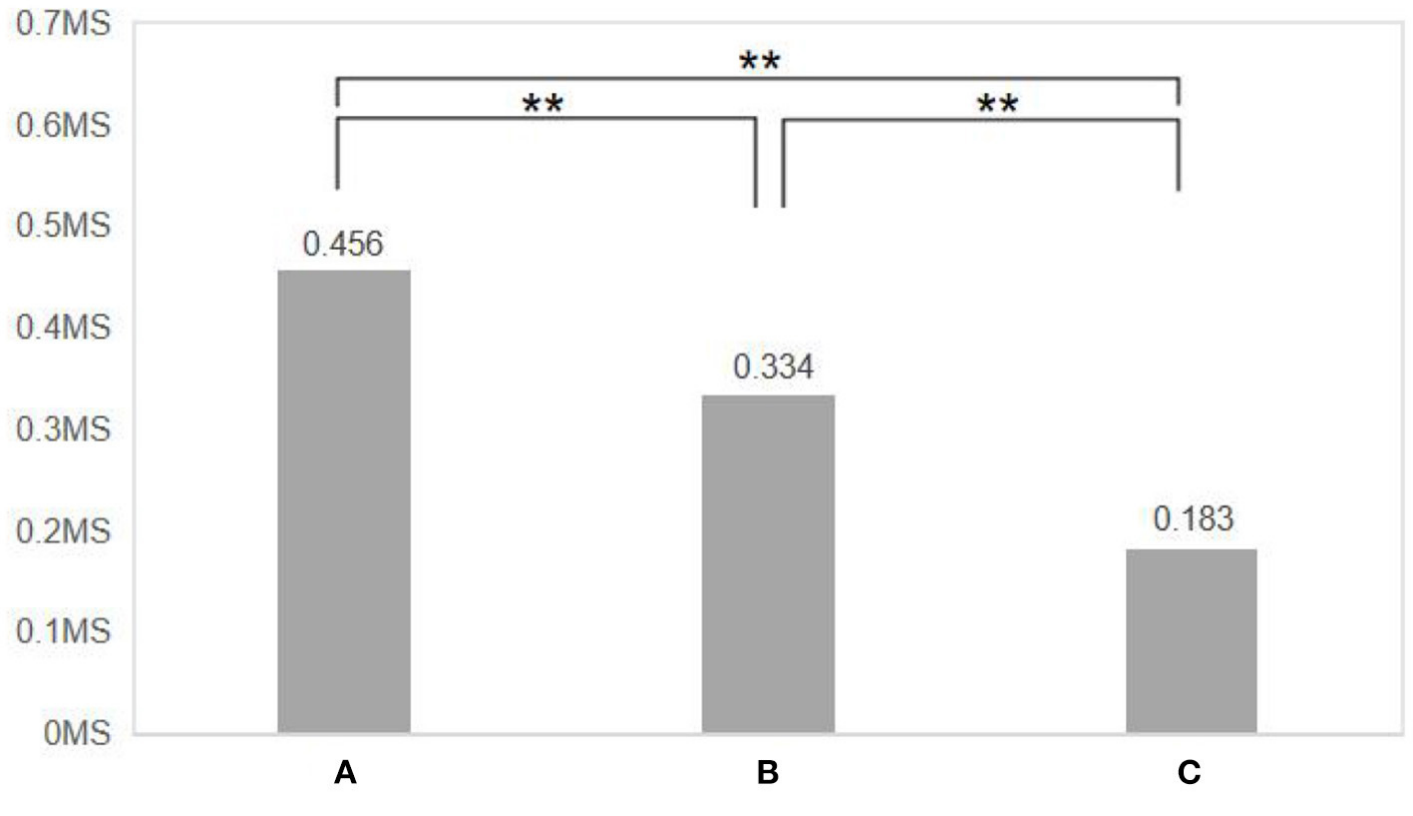
Comparison of the difference in tare values before and after the experience of three VR scenes. *N* = 48; mean ± SD; **P < 0.01; verified by paired *t*-test.

The greater the difference in the increase in skin electrical values, the greater the effect of the virtual experiment scenario on the positive emotions of the subjects. One-way analysis of variance (ANOVA) was used to further analyze the difference in skin electrical variation obtained from the three virtual scenarios, and after Bonferroni multiple comparisons Figure 8, it was found that the difference in skin electrical variation obtained from the three virtual scenarios was significantly different (*F* = 16.28, *d.f*. = 2, *P* = 0.000), with the highest rate of skin electrical variation in scenario A, the second highest in scenario B, and the smallest in scenario C. the second highest and scene C the smallest. This indicates that the residential compound space with the right amount of fitness and exercise facilities has a better promotion effect on residents' emotional health under the same green view rate, while the scenario A with a higher green view rate has a better promotion effect on residents' emotional health compared to the scenario C with the same amount of fitness and exercise facilities, and the comparison between scenario B and scenario C reveals that even the scenario B without fitness facilities has a significantly higher promotion effect on residents' emotional health than the scenario C. The comparison between Scenario B and Scenario C shows that even Scenario B without fitness facilities has a significantly higher effect on residents' emotional health than Scenario C.

### Differences in the effects of VR experimental scenarios on the emotional health of people of different genders

**Figure 9:**
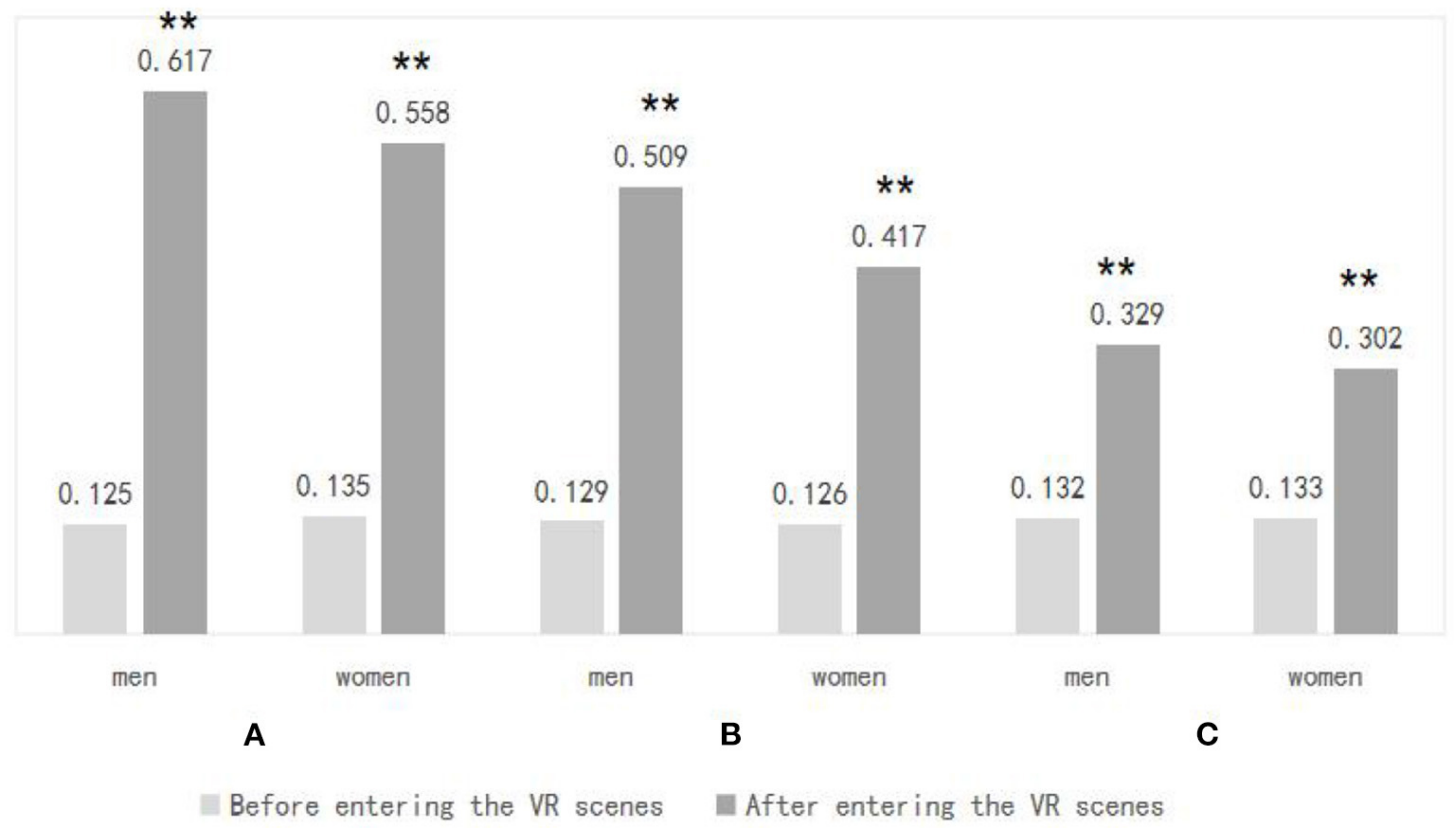
Skin electrical data of different genders were compared after entering three VR environments. *N* = 48; mean ± SD; **P < 0.01; verified by paired *t*-test.

As shown in Figure 9, subjects of different genders showed significant changes in their skin electrical values after the experience of the three virtual experimental scenarios. In Scenario A, the skin electrical values of males were 0.125 ± 0.063 before and 0.617 ± 0.382 after the experience (*T* = −5.978, *d.f*. = 22, *p* = 0.000), and the skin electrical values of females were 0.135 ± 0.059 before and 0.558 ± 0.157 after the experience (*T* = −8.468, *d.f*. = 24, *P* = 0.000); in scenario B, the electrodermal values for males were 0.129 ± 0.059 before and 0.508 ± 0.157 after the experience (*T* = −10.854, *d.f*. = 22, *P* = 0.000) and for females in the pre-experience and 0.126 ± 0.056 post-experience, and 0.417 ± 0.156 post-experience (*T* = −8.726, *d.f*. = 24, *P* = 0.000); in scenario C, men had 0.132 ± 0.059 pre-experience and 0.329 ± 0.167 post-experience (*T* = −5.809, *d.f*. = 22, *P* = 0.000), and in females the electrodermal values were 0.133 ± 0.042 before the experience and 0.302 ± 0.173 after the experience (*T* = −4.537, *d.f*. = 24, *P* = 0.000). This indicates that the three virtual experimental scenarios had a promotional effect on emotional well-being in subjects of different genders.

**Figure 10:**
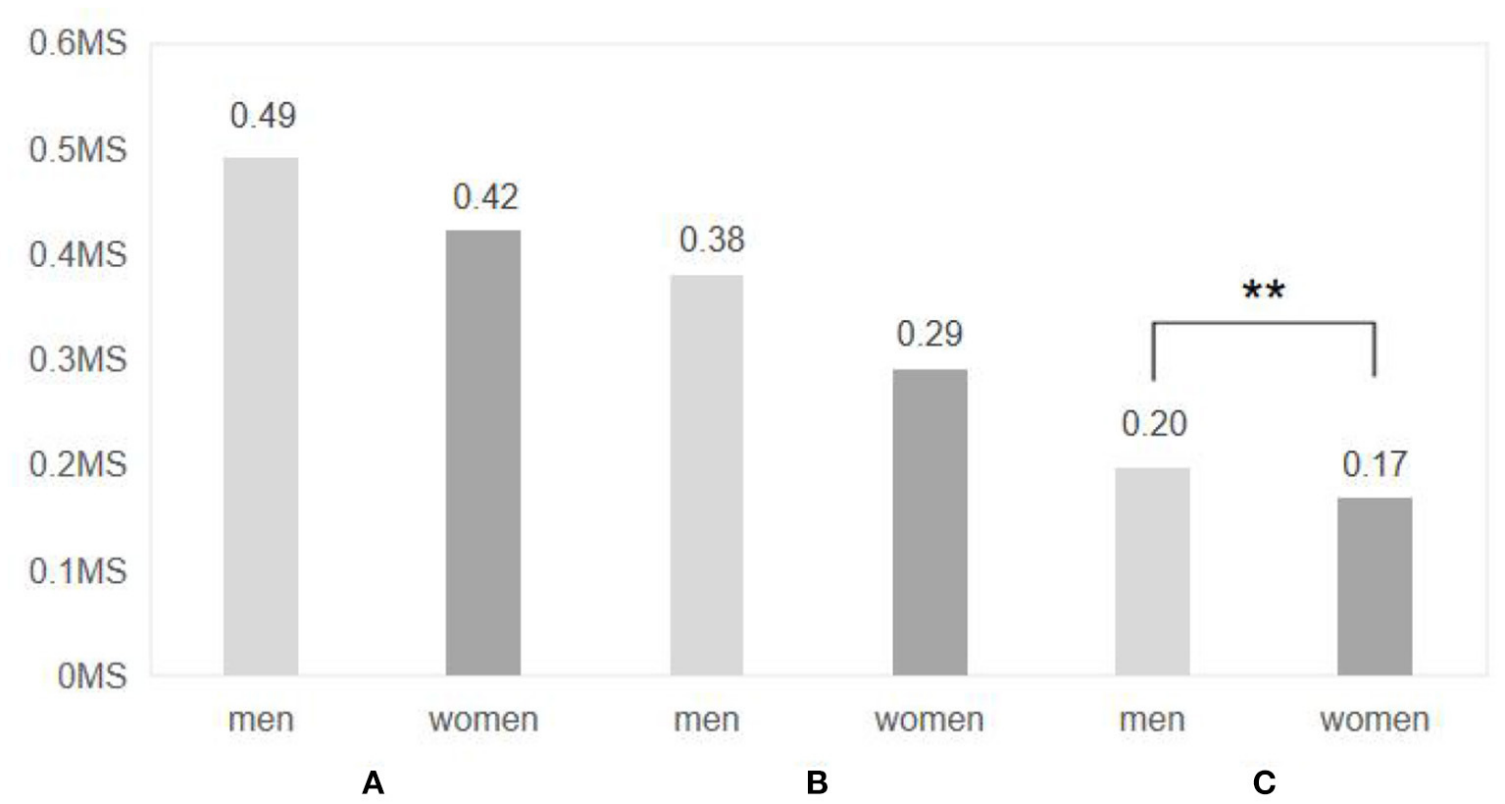
Comparison of electrodermal difference between different genders after entering three virtual Spaces. *N* = 48; mean ± SD; **P < 0.01; verified by ANOVA.

By comparing the electrodermal differences between subjects of different genders before and after experiencing the three virtual experimental scenarios (Figure 10), it was found that only in Scenario C were the data significantly different between males and females (0.197 ± 0.159 for males and 0.170 ± 0.183 for females, *p* = 0.047), indicating that in the scenario of a residential courtyard space with a lower rate of green vision, the emotional facilitation was significantly higher for males than for females. At the same time, male subjects in Scenario A and Scenario B also had slightly greater skin electrical differences than female subjects (Scenario A: 0.492 ± 0.385 for males and 0.424 ± 0.245 for females; Scenario B: 0.380 ± 0.164 for males and 0.291 ± 0.163 for females), suggesting that the residential courtyard space scenario had a greater emotional facilitation effect on males.

### Differences in the effects of VR experimental scenes on the emotional health of different age groups

**Figure 11:**
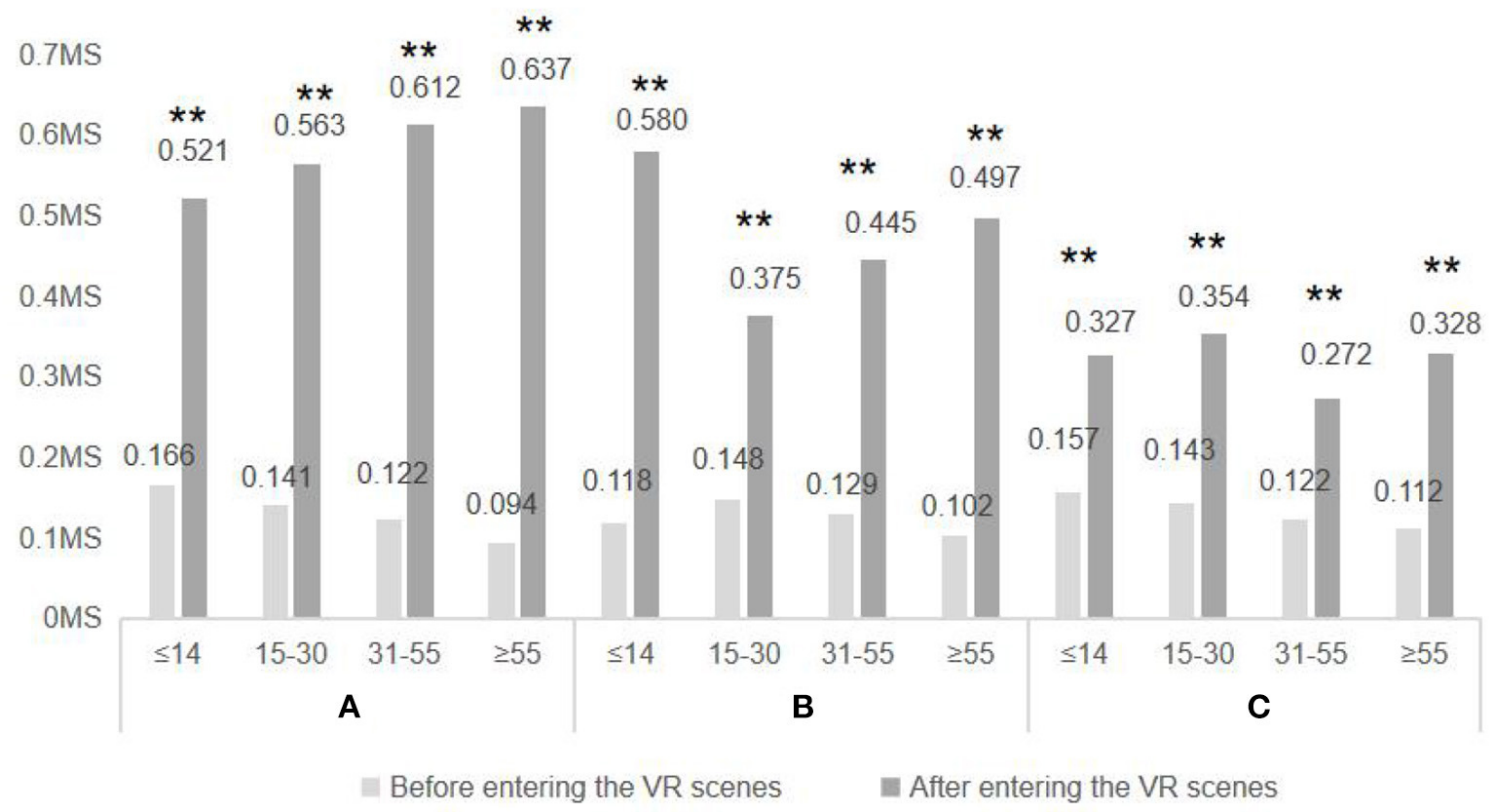
Skin electrical data of different age were compared after entering three VR environments. *N* = 48; mean ± SD; **P < 0.01; verified by paired *t*-test.

As shown in Figure 11, subjects of different ages experienced a significant increase in skin electrical values after the three VR experimental scenes, which indicates that the three VR experimental scenes had a promotional effect on emotional health for subjects of different ages. Specifically, in Scenario A, the difference in skin electrical values before and after the experiment increased with age, suggesting that the effect of courtyard space with an appropriate amount of trees and shrubs and sports facilities on the emotional health promotion of the population increased with age; in Scenario B, the difference in skin electrical values before and after the experiment was significant in the population under 15 years old (0.468) and over 55 years old (0.395), while the difference was smaller in the population aged 15–30 years old (0.227) and 31–55 years old (0.216). This suggests that courtyard spaces with a good level of greenery but without exercise facilities have a greater mood boosting effect on children, adolescents and older adults, and a smaller mood boosting effect on the young adult population; in Scenario C, the changes in subjects' skin electrical value differences before and after the experiment were not significant, with In Scenario C, there was little change in the skin electrical value difference before and after the experiment, with slight changes before and after the scenario experience for the 15–30 (0.211) and 55+ (0.216) age groups, and almost no change for the under −15 and 31–55 age groups, suggesting that courtyard spaces with a low level of greenery (even if they provided a moderate amount of exercise facilities) had some effect on the mood boosting effect for the 15–30 and 55+ age groups, but not for the other groups.

**Figure 12:**
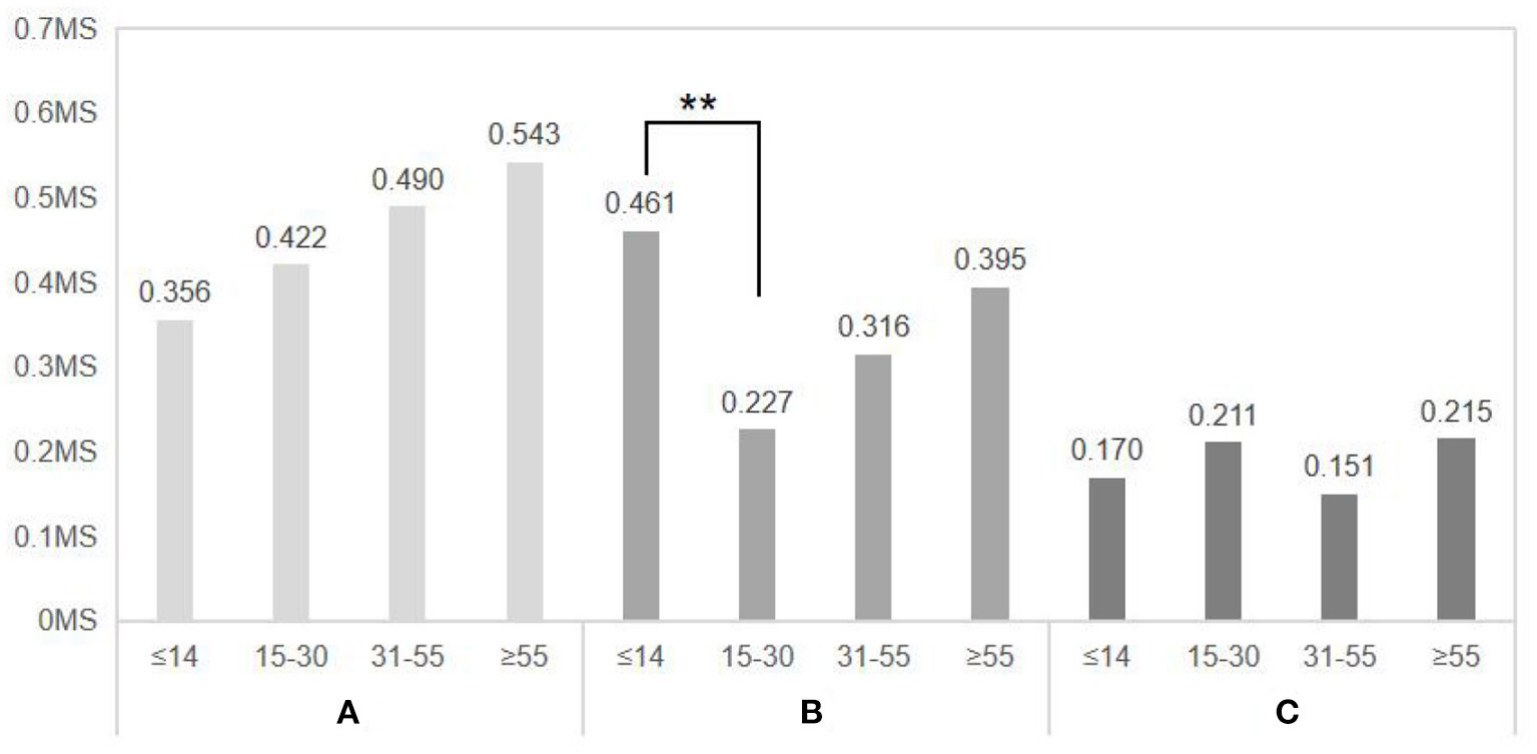
Comparison of the difference in skin electricity after entering three virtual spaces for subjects of different ages. *N* = 48; mean ± SD; **P < 0.01; verified by ANOVA.

As shown in Figure 12, a one-way ANOVA revealed that the values of electrodermal changes were significantly higher in subjects ≤ 14 years old than in subjects 15–30 years old when experiencing virtual experimental scenario B (≤ 14 years old 0.461 ± 0.180, 15–30 years old 0.227 ± 0.146, *p* = 0.006), and subjects ≤ 14 years old did not perceive the absence of fitness facilities significantly and were able to obtain a higher emotional impact than subjects aged 15–30 years. In the virtual experimental scenarios A and C, the analysis showed no significant differences between groups (scenario A: *F*= 0.595, *d.f*. = 3, *p* = 0.622; scenario C: *F* = 0.415, *d.f*. = 3, *p* = 0.743), but the difference in electrodermal values was significantly higher in scenario A than in scenario C, suggesting that scenarios with a higher rate of greenness had a greater effect on subjects' emotions.

## Discussion

### Residential courtyard space promotes population emotional health - VR identification of landscape elements

VR technology has gained increasing recognition in urban environmental cognition and experience research, and some scholars have demonstrated the feasibility of the technology in environmental health design research (14, 15), but some scholars have recently argued that research findings in virtual natural environments are unreliable and need to be further calibrated and validated in the future (37). This study explored the effects of two landscape elements: green vegetation and fitness facilities, on the emotional health of people in residential compound space based on a virtual experimental platform, and the results showed that the residential compound space environment with a higher green view rate has a better effect on the emotional health of people, which is consistent with the previous study conducted in a real environment, which concluded that “the magnitude of the green view rate is positively correlated with emotional recovery” (38, 39) and “higher recovery is more likely to be associated with natural rather than artificial landscape elements” (40). This study confirms that VR technology could be similar to the real environment in terms of environmental visual perception, and has the advantage of better controllability and manipulation in terms of experimental methods.

In addition, virtual experimental scenarios are also highly actionable and immersive, which allows elements of the virtual environment to be perceived more accurately. Studies have shown that an increase in the number of fitness facilities could help motivate residents to generate more physical activity, which in turn promotes emotional well-being (41, 42). Although people could not realistically touch fitness facilities and perform physical activity in virtual environments, previous studies based on low vs. high quantity and quality of green spaces have confirmed the positive effect of perceived green space use factors on population mood improvement (43–45). In this study, only three additional fitness facilities were added to Scene A compared to Scene B. The results indicated that the subjects received higher emotional health promotion in Scene A. This again confirms that the fitness facility, a landscape element in the VR environment, could be perceived by participants as a potential physical activity possibility and consequently generate motor associations. We were surprised to find that virtual environments could provide imagination for users to generate creative ideas in response to changes in environmental states and interaction behaviors (46), producing emotional health effects that are close to those in real environments.

### Gender perception differences—green vegetation and fitness facilities in residential courtyard spaces

There is a consensus that gender influences people's perception and use of urban public space (47–49), but there has not been extensive discussion focusing on gender differences in the effects of residential compound space on the emotional health of people. The present study showed that the residential courtyard space environment under virtual environmental conditions was emotionally health-promoting for subjects of different genders, with men obtaining slightly better emotion-promoting effects than women in all scenarios, and this difference was more pronounced in Scenario C, where fitness facilities were the main landscape element.

Starting from green vegetation landscape elements, which is different from the previous findings of Richardson& Mitchell, who conducted a census of 2.86 million people and concluded that women are more happy with green space (50). Asa et al. found that women are better able to perceive all aspects of nature (e.g., species richness, vegetation density, etc.) through a mailed questionnaire (51), which generally supports the findings of Richardson & Mitchell's study. We analyzed that the difference in findings may be due to the type of vegetation, with “flowers” considered to be the most influential landscape element on women's emotional and mental state, while “trees and greenery” had a significant effect on men (52). The three virtual experimental scenarios constructed in this study were all green trees, shrubs, and lawns without colored flowers, and further comparative studies on the effects of green vegetation and colored flowers on the emotional health of the population can be conducted under virtual environment conditions.

Starting from the fitness facility landscape element, Schipperijn et al. found that women scored significantly higher than men in activities such as leisure, socializing, and walking in the real environment through a questionnaire (45), again inconsistent with the findings of our study. However, the participants in Schipperijn et al.'s study underwent complex environmental experiences in the real environment, especially leisure and socializing, whereas the subjects in the present study only roamed alone in the virtual experimental scenario and were not stimulated with other senses and behaviors besides visual perception, which may have contributed to the inconsistency of the findings.

### Age-perceived differences—green vegetation and fitness facilities in residential courtyard spaces

Age is also an important factor to be considered in the design of urban public spaces (45, 51, 53). Currently, academic research on the health effects of public spaces has focused more on the applicability of public spaces from a certain age group, and less on differentiated research for different age groups, and even less on the spatial environment of residential compounds. This study showed that under virtual environment conditions, people of all ages showed the best mood boosting effects in residential compound spaces with high green view rates and appropriate amount of fitness facilities, and there were no significant differences between groups, which is consistent with previous studies that reported that positive mood is more likely to be generated in places with good housing environments and sufficient green spaces (54). It is noteworthy that the values of electrodermal changes also tended to be equivalent in scenarios A and B with the same green vision rate for those ≤ 14 years old, suggesting that adolescents receive more positive emotional health effects in environments with higher green vision rates. This follows the same trend as the findings of Mireia et al. who noted a significant association between the daily green concentration to which adolescents are exposed and their daily mood, with exposure to natural environments enhancing positive mood (55).

In addition, our study found that people ≤ 14 years old received better mood boosting effects in residential compound spaces without fitness facilities, which seems contrary to the findings of some previous studies. The study by Asa et al. confirmed that the rhythm of people's lives changes with age and that lower levels of physical fitness lead to a shift in attention to less energetic activities, so that older people are more inclined to participate in nature-related activities, while younger people have a greater preference for physical activities (48). In-depth analysis revealed that the conclusions we obtained were not contradictory to previous research results: the fitness facilities we arranged in the virtual experimental scenario were common street activity equipment in the city, and these facilities brought low-intensity physical activities that were more in line with adult use needs, while some of the children and adolescents under 14 years old did not meet the physiological requirements for using fitness facilities, and they preferred playing ball, running, swimming, and other Therefore, they are less sensitive to the presence or absence of fitness facilities than other age groups.

## Conclusion

This study uses a precise quantitative approach to explore the impact of two landscape elements, vegetation and sports facilities, on the emotional well-being of people in residential courtyard spaces. The results of the study show that residential courtyard spaces with higher greenery are more effective in promoting emotional health (for both men and women, regardless of age group) than those with more fitness facilities, and that a moderate amount of fitness facilities in a high greenery environment could enhance the health promotion effect of the environment; residential courtyard spaces with fitness facilities as the main landscape element are more effective in promoting emotional health for men than for women; and residential courtyard spaces with greenery as the main landscape element are more effective in promoting emotional health for men than for women. The residential courtyard space with green vegetation as the main landscape element without fitness facilities has a slightly greater effect on the emotional health of men than women, and a greater effect on the emotional health of people under 14 years old than those over 14 years old. These findings provide a quantitative basis for the healthier design and modification of residential courtyard spaces for both green vegetation and fitness facilities. In the author's opinion, in the healthy construction of urban residential courtyard space, if funds are not too abundant, priority can be given to upgrading the level of greenery and reserving space for activities, and it is not recommended to set up fitness facilities in isolation in a space without landscape greenery.

This study confirms the applicability of VR technology in environmental health design research, which combined with wearable physiological sensor measurement technology, helps to better measure and provide feedback on the health benefits of environmental experiences for people, but there are still some limitations. (1) Although the latest Mars software provides real-time editing of materials and components, efficient and fast real-time rendering, and diverse expressions to more realistically and finely represent the real-life effects of the built environment, the simulation of both the experimental model and the experimental process need to be further improved, such as increasing the diversity of plant growth levels and enhancing the diversity of subjects during the environmental experience. (2) In order to control the variables, the indicators of the level of vegetation and sports facilities in this study focus on the dosage aspect. In fact, the types, colors and combinations of plants and the types and materials of sports facilities are all indicators of the level of vegetation and sports facilities, which also affect the environmental experience of people of different genders and ages in the residential courtyard space, and more indicators can be included in the study. (3) Through literature review and questionnaire survey, this study selects the more important landscape elements of vegetation and sports facilities for research, but there are many other landscape elements that affect people's emotional health in residential compound space, such as lighting and paving, and the mechanism of the influence of different landscape elements on people's emotional health is still unclear, so more comprehensive and systematic research should be conducted in the future. (4) The health benefits of the environment on people include three dimensions: promoting emotional health, enhancing physical activity and increasing social interaction. This study focuses on the single dimension of promoting people's emotional health by specific landscape elements in courtyard spaces, and further research can be conducted on the dimensions of enhancing physical activity and increasing social interaction. As the technology matures, the health benefits of more landscape elements in the urban living environment and their impact mechanisms will be explored in the future based on the virtual reality experimental platform, so that more residents could enjoy the health benefits brought by the environment.

## Data availability statement

The raw data supporting the conclusions of this article will be made available by the authors, without undue reservation.

## Ethics statement

The studies involving human participants were reviewed and approved by College of Landscape Architecture, Sichuan Agricultural University, China. The patients/participants provided their written informed consent to participate in this study.

## Author contributions

EF: conceptualization, funding acquisition, supervision, methodology, and writing—original draft. JZ: formal analysis, writing—review and editing. YR: statistical analysis and writing—review and editing. XD: formal analysis, writing—review and editing. XinL and XiL: validation and writing—review and editing. All authors contributed to the article and approved the submitted version.

## Conflict of interest

The authors declare that the research was conducted in the absence of any commercial or financial relationships that could be construed as a potential conflict of interest.

## Publisher's note

All claims expressed in this article are solely those of the authors and do not necessarily represent those of their affiliated organizations, or those of the publisher, the editors and the reviewers. Any product that may be evaluated in this article, or claim that may be made by its manufacturer, is not guaranteed or endorsed by the publisher.
